# Methanol Extract of *Thottea siliquosa* (Lam.) Ding Hou Leaves Inhibits Carrageenan- and Formalin-Induced Paw Edema in Mice

**DOI:** 10.3390/molecules29204800

**Published:** 2024-10-11

**Authors:** Aneeta Renny, Jameema Sidhic, Alby Tom, Aswathi Moothakoottil Kuttithodi, Joice Tom Job, Rajakrishnan Rajagopal, Ahmed Alfarhan, Arunaksharan Narayanankutty

**Affiliations:** 1 Division of Cell and Molecular Biology, PG & Research Department of Zoology, St. Joseph’s College (Autonomous), Calicut (Affiliated to University of Calicut) 673008, India; aneetarenny@gmail.com (A.R.); albytom@devagiricollege.org (A.T.); abcaswathi@gmail.com (A.M.K.); 2 Phytochemistry and Pharmacology Division, PG & Research Department of Botany, St. Joseph’s College (Autonomous), Calicut 673008, India; jameemasidhic@gmail.com; 3 Department of Botany and Microbiology, College of Science, King Saud University, P.O. Box 2455, Riyadh 11451, Saudi Arabia; rrajagopal@ksu.edu.sa (R.R.); alfarhan@ksu.edu.sa (A.A.)

**Keywords:** anti-inflammatory activity, carrageenan, acute inflammation, chronic inflammation, ethnomedicinal, *Thottea siliquosa*, paw edema

## Abstract

Inflammation is a physiological condition that when unattended causes serious health concerns over the long term. Several phytocompounds have emerged as promising sources of anti-inflammatory agents. *Thottea siliquosa* is a traditional medicine for inflammatory and toxicity insults; however, this has not been scientifically confirmed. The purpose of this study is to evaluate the anti-inflammatory properties of *T. siliquosa* methanol leaf extract in a mouse model. This study investigates the anti-inflammatory activities of a plant extract obtained from leaves of *T. siliquosa* (TSE) with a focus on carrageenan- and formalin-induced paw oedema in mice. The extract’s efficacy was assessed using well-established inflammation models, and the results showed a considerable reduction in paw edema in both cases. In the case of carrageenan model TSE at 50 mg/kg showed a 53.0 ± 2.5% reduction in edema, while those treated with TSM at 100 mg/kg exhibited a 60.0 ± 1.8% reduction (*p* < 0.01). In the case of a formalin model when a higher dose of TSE (100 mg/kg) was given, paw thickness decreased by 47.04 ± 1.9% on the fifth day and by 64.72 ± 2.2% on the tenth day. LC-MS analysis reported the presence of gallic acid, quinic acid, quercetin, clitorin, myricitrin, retronecine, batatasin II, gingerol, and coumaric acid in the extract. Overall, this study confirms that *T. siliquosa* extract exerts anti-inflammatory effects in animals and is possibly mediated through the combined effects of these phytochemicals.

## 1. Introduction

Inflammation is essential for maintaining homeostasis, which is triggered by the immune system in an instance of tissue damage or injury, and this reaction ensures tissue survival [1]. Prolonged inflammation significantly hinders wound healing and contributes to severe wounds and chronic inflammation [2]. Switching the duration of inflammation from short to long adversely affects normal body physiology and leads to an increased incidence of various non-communicable diseases in individuals, irrespective of their age [3]. There are numerous immunomodulatory medications on the market, including signaling pathway inhibitors, non-steroidal anti-inflammatory medicines, immunosuppressants, and corticosteroid [4,5,6]. These anti-inflammatory medications are associated with adverse effects. Therefore, there is a growing tendency to use natural anti-inflammatory components to maximize pharmacological effectiveness while minimizing unwanted side effects [7]. Modern allopathic medications are often composed of single molecules; however, herbal remedies contain a range of chemicals that collaborate on intricate biological pathways, and in comparison with isolated pure drugs, most of the time, they show higher activity against diseases [8].

Pain and inflammation continue to be the most difficult and debilitating health issues, affecting 80% of adults globally, even in the presence of adequate medication [9]. The most common condition that causes both physical damage and psychological issues is untreated, protracted pain [10]. Indigenous cultures have long used medicinal plants, and this knowledge is valuable because it supports the conservation of plant species and ecosystems, helps maintain cultural heritage, offers essential resources for community healthcare, and aids in the discovery and development of new medications [11]. Plants have been used medicinally for ages, and many contemporary medications are made from plant components. Digoxin, a heart treatment, is produced from foxglove [12], whereas artemisinin, an antimalarial drug, is derived from sweet wormwood [13]. Developing novel plant-based medications entails isolating active chemicals, performing rigorous clinical studies, and assuring safety and efficacy, which can result in effective therapies with fewer side effects than synthetic pharmaceuticals.

Despite the fact that medicinal plants have been used extensively and for a long time in complementary and alternative medicine, some questions have been raised about their safety [14]. Traditional knowledge about medicinal plants and their use by indigenous cultures is important for several reasons. In many regions of the world, traditional therapies have been practiced for generations. Because they are inexpensive and sustainable, herbal remedies are growing in popularity [15,16]. *Thottea* is one such genus, with a lot of pharmacological properties [17,18]. *Thottea* genus comprises about 25 species that are distributed across the southeast Asian countries. This genus is rarely found in tropical lowland forests and can be located at altitudes up to approximately 1000 m [19]. The species *T. siliquosa* is one of the species that is less explored for its biological activity. Some studies have explored its effectiveness against inflammation under in vitro conditions [17,20]. However, no research has explored these biological activities in an animal model. The lack of knowledge on the toxicity and safety of many therapeutic plants reduces confidence in herbal treatment. In order to avoid negative effects and fatalities, it is crucial to assess the toxicity and safety of herbal treatments used to treat a variety of conditions [14,21].

Hence, this study aims to evaluate the anti-inflammatory activity of *T. siliquosa* in an animal model of both acute and chronic inflammation. In silico analysis was also carried out to identify the bioactive metabolite present in the *T. siliquosa* extract and explain the possible mechanism of action.

## 2. Results

### 2.1. Qualitative Estimation of Phytochemicals in T. siliquosa

**Figure 1:**
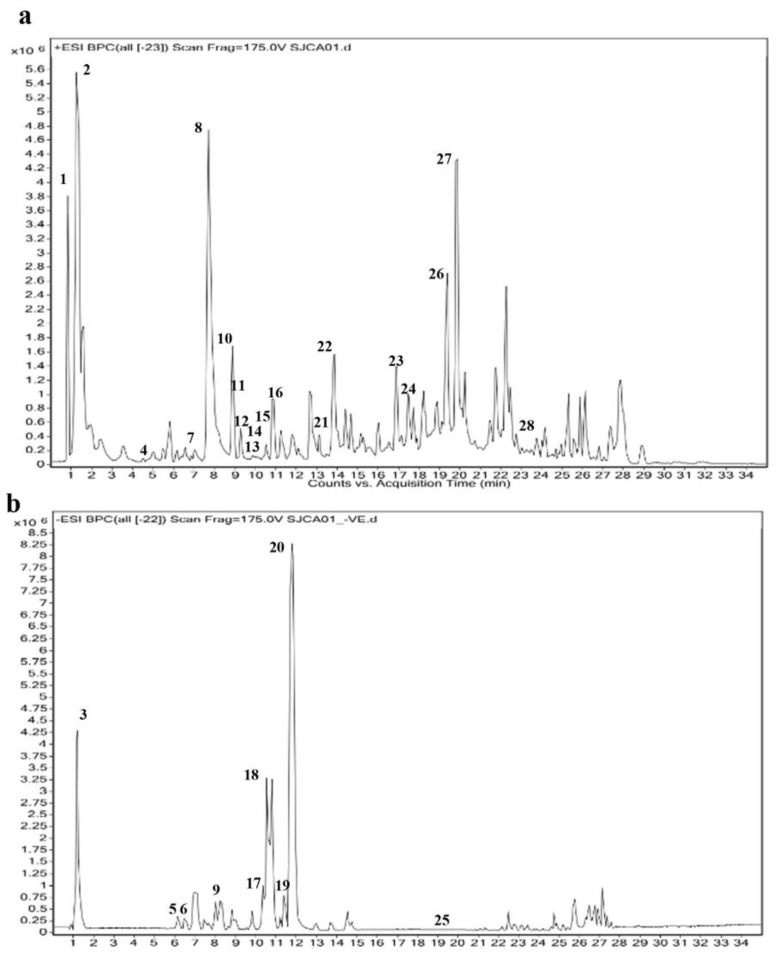
Total ion chromatogram obtained by LC-MS of TSE: (**a**) chromatogram in the positive ionization mode; (**b**) chromatogram in the negative ionization mode. The numerical 1–28 indicates the compound numbers in Table 1.

The extract indicated the presence of several phenolic and flavonoid compounds. Gallic acid, resorcinol, quinic Acid, clitorin, myricitrin, retronecine, batatasin II, gingerol, and coumaric acid were all detected using LC-MS. The detailed results are depicted in Figure 1a,b and Table 1. Supplementary Figure S1 describes the MS spectra and MS/MS spectra of individual compounds identified. The structures of major compounds are also included in Supplementary Figure S2.

### 2.2. In Silico Anti-Inflammatory Activity

**Figure 2:**
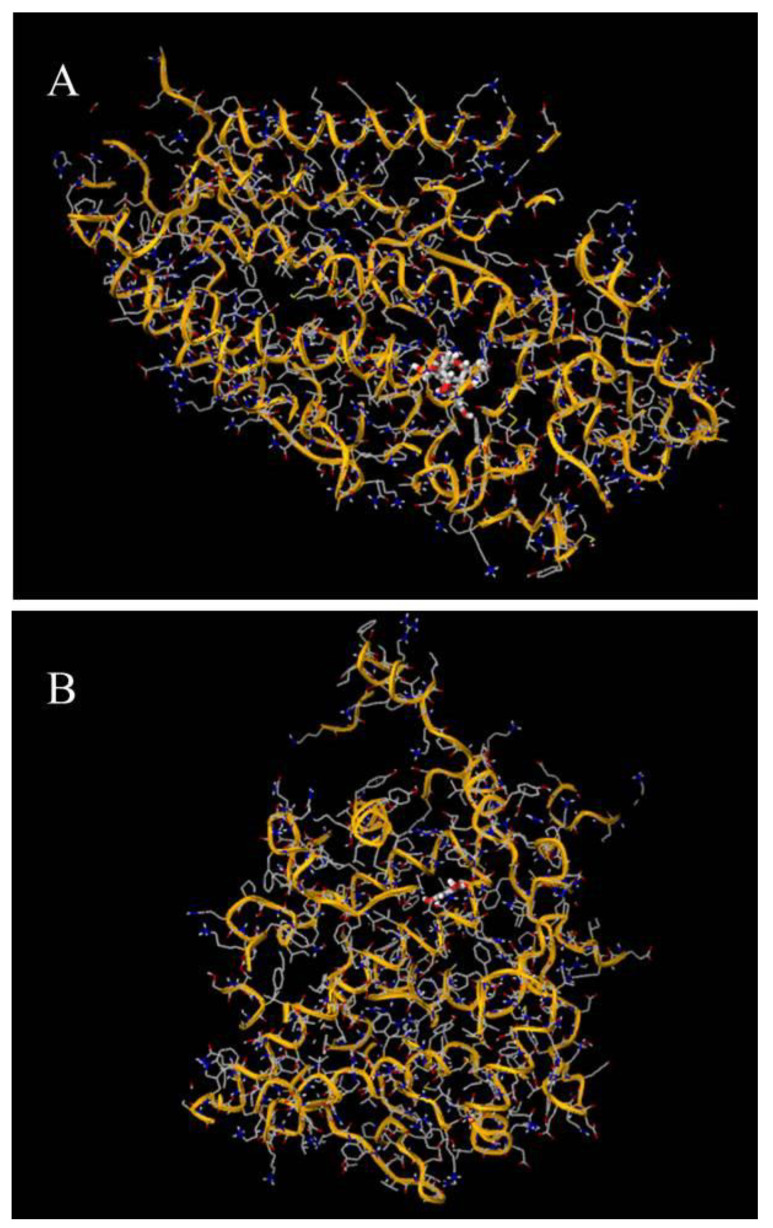
Docking poses of quercetin with lipoxygenase (**A**) and cyclooxygenase (**B**).

Molecular docking analysis revealed the interaction of various compounds against lipoxygenase and cyclooxygenase enzymes. The most active one was quercetin (Table 2) and the least active was quercitrin (Figure 2).

Molecular docking analysis revealed the interaction of various compounds against lipoxygenase and cyclooxygenase enzymes. The most active one was quercetin (Table 2) and least active was quercitrin (Figure 2). The inhibitory effects of various compounds on LOX (lipoxygenase) and COX-2 (cyclooxygenase-2) were assessed and expressed as binding affinities (in kcal/mol). For LOX inhibition, diclofenac demonstrated the strongest inhibitory effect with a binding affinity of −7.200 ± 0.61 kcal/mol. This was followed closely by myricitrin with −7.075 ± 0.22 kcal/mol and quercetin with −6.625 ± 0.31 kcal/mol, both showing strong inhibitory potential. (-)-Epicatechin also displayed significant activity with affinities of −6.475 ± 0.30 kcal/mol. In contrast, compounds like quercitrin (−5.150 ± 0.07 kcal/mol) and ketosantalic acid (−4.975 ± 0.21 kcal/mol) demonstrated relatively weaker LOX inhibition (Table 2).

Regarding COX-2 inhibition, diclofenac again exhibited the strongest inhibitory activity with a binding affinity of −8.125 ± 0.27 kcal/mol, followed by quercetin (−7.875 ± 0.56 kcal/mol) and gallic acid (−7.400 ± 0.29 kcal/mol), indicating substantial activity. Myricitrin also showed strong COX-2 inhibition with −7.475 ± 0.20 kcal/mol. (-)-Epicatechin also exhibited moderate inhibition with affinities of −6.625 ± 0.31 kcal/mol. These results suggest that compounds like quercetin and myricitrin possess potent dual inhibitory activity against both LOX and COX-2, making them promising candidates for anti-inflammatory therapy (Figure 2).

### 2.3. Anti-Inflammatory Activity in Acute Model

There was no observable toxicity symptoms or mortality in animals administered with different doses of TSE (1 and 2 g/kg). Further, no significant variation was noted in body weight (initial and final) or food and water consumption in comparison with animals in the control group.

**Figure 3:**
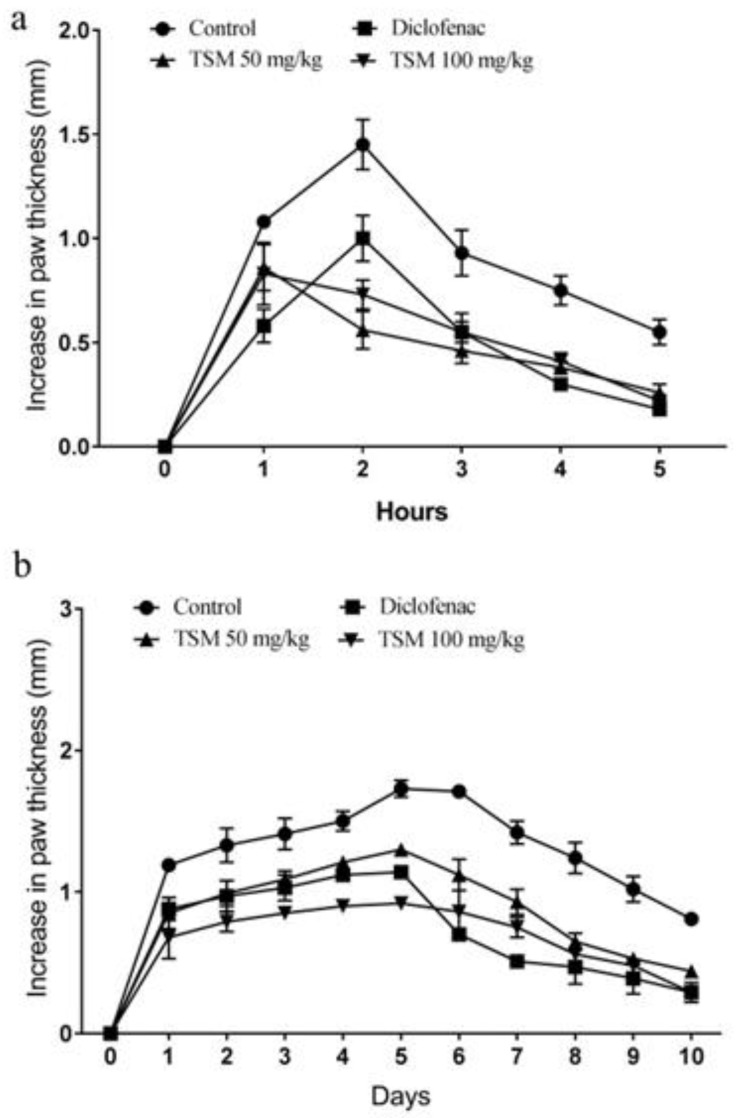
Changes in the paw thickness of mice under different treatment groups in carrageenan-induced acute (**a**) and formalin-induced chronic (**b**) models.

During the carrageenan-induced acute paw edema, the initial paw thickness increased significantly from 1.78 ± 0.18 mm to 2.33 ± 0.29 mm by the fifth hour (Figure 3a). Treatment with the standard drug diclofenac (10 mg/kg) resulted in a 67.3 ± 2.3% reduction in paw edema by the fifth hour after carrageenan administration (Table 3). Animals treated with TSE at 50 mg/kg showed a 53.0 ± 2.5% reduction in edema, while those treated with TSM at 100 mg/kg exhibited a 60.0 ± 1.8% reduction (*p* < 0.01).

### 2.4. Anti-Inflammatory Activity in the Chronic Model

Paw thickness increased significantly with the application of formalin; it peaked on day five at 3.78 ± 0.17 mm, a rise from 2.05 ± 0.12 mm at the beginning. Paw thickness dropped to 2.86 ± 0.08 mm by the tenth day. After formalin injection, the conventional medication diclofenac decreased paw thickness by 46.17 ± 2.1% on day five and by 76.89 ± 2.8% on day ten. After a low dose of TSE (50 mg/kg), paw thickness dropped by 24.74 ± 3.2% on the fifth day and 50.48 ± 2.3% on the tenth day. When a higher dose of TSE (100 mg/kg) was given, paw thickness decreased by 47.04 ± 1.9% on the fifth day and by 64.72 ± 2.2% on the tenth day (Figure 3b, Table 4).

## 3. Discussion

Pain and inflammation are hallmark symptoms of a wide range of illnesses that significantly impacting overall health and well-being [22]. These responses are the body’s natural defense mechanisms against injury, infection, or harmful stimuli, and while they play an essential role in healing, prolonged or excessive pain and inflammation can lead to detrimental outcomes. Many diseases, including autoimmune disorders, infections, and chronic conditions like arthritis or cardiovascular diseases, are associated with persistent pain and inflammation.

When inflammation becomes chronic, it can contribute to a cascade of negative health effects. It often results in physical discomfort, reduced mobility, and impaired function, which can lower quality of life. In both humans and animals, chronic pain is linked to emotional distress, fatigue, weakness, and, in severe cases, depression. This emotional burden can exacerbate physical symptoms, creating a vicious cycle that further deteriorates overall health. Moreover, prolonged inflammation is associated with increased risks of developing additional complications, such as organ damage, heart disease, or cancer, which can shorten life expectancy [23,24].

Usually, the treatment regimen involves the use of various classes of analgesics and anti-inflammatory drugs [25]. Various studies state that the administration of these drugs is associated with various health problems [26]. Hence, there is a paradigm shift towards using herbal remedies instead of modern medicines [27,28].

In this work, the in silico and in vivo anti-inflammatory efficacy of *T. siliquosa* methanolic leaf extract was analyzed against carrageenan- and formalin-induced inflammation [29,30,31,32,33,34]. These nociceptive signals result in the release of several inflammatory mediators, including histamines, prostaglandins, and cytokines, which contribute to discomfort and swelling in the affected region [35,36,37,38,39]. TSE had an inverse dose-dependent anti-inflammatory impact on acute and chronic paw edema in mice. The reduction in inflammation could be attributed to TSE’s phytochemical component(s) against carrageenan- and formalin-induced inflammatory mediators. LC-MS analysis revealed the presence of phenolic acids and flavonoid glycosides in TSE, which have the ability to prevent certain inflammation by preventing immune cell activation [40,41,42,43,44]. The response is also driven by a cascade of enzymatic reactions of cyclooxygenase (COX) and lipoxygenase (LOX) pathways, which are key enzymes in the inflammatory process [45,46]. Additionally, the recruitment of immune cells to the site of inflammation leads to the release of oxygen-derived free radicals and nitric oxide (NO), both of which further exacerbate the inflammatory response [47]. Free radicals—such as superoxide anions—and NO are known to cause oxidative stress and tissue damage, intensifying the severity of the edema. Nitric oxide, produced primarily by inducible nitric oxide synthase (iNOS), plays a dual role by contributing to both vasodilation and cytotoxicity, further complicating the inflammatory milieu [48]. The synergistic effect of these mediators, along with prostaglandins, results in sustained inflammation and tissue damage, making carrageenan-induced inflammation a robust model for studying the mechanisms underlying inflammation and testing the efficacy of anti-inflammatory drugs [49]. Therefore, this model remains a cornerstone in inflammation research, providing valuable insights into the role of various biochemical pathways in the inflammatory process. In previous studies, the *T. siliquosa* has been shown inhibit cellular inflammation via modulating pro-inflammatory cytokine production [50,51]. HR-LC-MS/MS analysis revealed the presence of a diverse range of phytochemicals, including gallic acid, quinic acid, quercetin, clitorin, myricitrin, retronecine, batatasin II, gingerol, and coumaric acid. These compounds are well known for their broad spectrum of biological activities, particularly their anti-inflammatory properties [52]. Each of these phytochemicals has been shown to exert therapeutic effects independently or synergistically, thereby contributing to their potential efficacy in managing inflammation [53]. For instance, gallic acid is a potent antioxidant with notable anti-inflammatory effects, primarily by inhibiting pro-inflammatory cytokine production [54]. Similarly, quercetin, a flavonoid, is well documented for its ability to suppress inflammation by reducing oxidative stress and modulating inflammatory signaling pathways [55]. Myricitrin, another flavonoid, has been reported to have strong antioxidant and anti-inflammatory properties, which help mitigate the immune response [56]. Furthermore, the extract is rich in flavonoids, which are a class of polyphenolic compounds known for their substantial role in suppressing inflammation. These flavonoids inhibit the production of key pro-inflammatory cytokines, including TNF-α (Tumor Necrosis Factor-alpha), IL-1 (Interleukin-1), IL-6 (Interleukin-6), IL-17 (Interleukin-17), and IFN-γ (Interferon-gamma). These cytokines are typically activated through various signaling pathways, most notably the NF-κB (Nuclear Factor kappa-light-chain-enhancer of activated B cells) pathway, which plays a central role in regulating the immune response to inflammation [57]. By inhibiting these inflammatory cytokines and signaling pathways, the phytochemicals help to dampen the inflammatory response, reduce tissue damage, and promote healing, highlighting the therapeutic potential of the extract in managing inflammatory conditions. Compounds including gallic acid suppress cellular inflammatory signaling [58]. Pro-inflammatory cytokines such as IFN-γ and TNF-α have role in controlling COX-2 and lipoxygenase (LOX) activity [59].

In silico analysis confirmed the individual roles of bioactive secondary metabolites such as myricitrin in inhibiting LOX and COX-2. LOX and COX-2 are important in triggering inflammatory cascades via arachidonate [60,61]. Previous research has shown that CD28 surface receptor activation of NF-kappa B needs reactive oxygen generation by 5-lipoxygenase [62]. Hence, a possible mechanism of inhibition of inflammation by TSE might be due to the blockade of NF-κB activation and subsequent inhibition of pro-inflammatory cytokine production and COX/LOX dual inhibition.

To explain the anti-inflammatory activity of the *T. siliquosa,* a previous study by Tom et al. [20] indicated a reduction in TLR4 expression in macrophages challenged with lipopolysaccharide. Toll-like receptors are known to induce inflammatory cytokine release mediated through NF-κB [63]. Furthermore, it has been demonstrated that flavonoids like quercetin suppress LPS-induced TLR4 signaling. In particular, quercetin decreases TLR4 expression and stops NF-κB from moving to the nucleus in human PBMCs and macrophages, which lowers inflammation [64]. These findings are supported by our LC-MS study, which verified the presence of quercetin. Modulating inflammatory signaling pathways is crucial for the treatment of inflammatory disorders. The NF-κB and TLR4 pathways can be activated by external stimuli, which results in the release of inflammatory cytokines and an inflammatory response [65,66].

It has also been established that oxidative stress contributes to inflammation. Antioxidants can assist in lowering inflammation [66,67]. Phytochemical analysis of leaf methanolic extracts of *T. siliquosa* revealed the presence of flavonoids and phenols. TSE phytochemicals reduce inflammatory responses with their antioxidant properties [68,69]. The high antioxidant capacity of this plant [50] can be attributed to alleviating paw edema. During inflammation, ROS generation might result in more deleterious conditions. TSE’s antioxidant properties can help to suppress the oxidative stress-related progress of the inflammation. Studies with several other plant remedies have good anti-inflammatory properties for extract with good antioxidant capacity, too [70]. The extract consists of various phytochemicals reported with antioxidant and anti-inflammatory properties, which are thought to be responsible for the extracts’ anti-inflammatory actions.

## 4. Materials and Methods

### 4.1. Extraction of T. siliquosa Leaves and Chemical Composition

*T. siliquosa* plants were obtained from Kozhikode, Kerala, India. The extraction was carried out as in our previous studies [20], and *T. siliquosa* extract (TSE) was suspended in 0.5% propylene glycol for in vivo analyses. LC-MS analysis was carried out according to previous methods [71].

### 4.2. In Silico Screening of Anti-Inflammatory Activity

The anti-inflammatory activity of selected major compounds was carried out using molecular docking. The compound structure was obtained from the PubChem database (https://pubchem.ncbi.nlm.nih.gov) and the structure of anti-inflammatory target proteins was obtained from RCSB Protein Data Bank (https://www.rcsb.org). Molecular docking was carried out using Mclue and Autodock as per previously described methods [50].

### 4.3. In Vivo Anti-Inflammatory Effect of TSE

Female Swiss albino mice weighing 23–29 g were maintained under standard husbandry conditions. Acclimatization of these animals was carried out for one week, and all experiments were approved by IAEC, Amala Cancer Research Centre (149/PO/Rc/S/99/CPCSEA) under permission number ACRC/IAEC/20(1) P5.

### 4.4. Animal Model Study

#### 4.4.1. Acute Toxicity Analysis of TSE

The oral acute toxicity of TSE was carried out using female Swiss albino mice according to OECD Test guideline 423. The animals were observed for 14 days for any sign of toxicity or mortality.

#### 4.4.2. Effect of TSE on Carrageenan-Induced Acute Inflammation

Female Swiss albino mice were separated into four groups, each with six individuals. Group I received only dextran and acted as the control. Group II was given diclofenac at a dose of 10 mg/kg as the standard reference medication. Groups III and IV received oral TSE dosages of 50 mg/kg and 100 mg/kg, respectively, for five days. On the fifth day, one hour after the drug’s final dose, 0.2 mL of a 1% carrageenan suspension in 0.1% carboxymethyl cellulose was injected into the subplantar area to cause acute inflammation. Paw thickness was measured using Vernier calipers and recorded at hourly intervals over five hours. The protocol was adapted from different studies.

#### 4.4.3. Formalin-Induced Chronic Inflammation

Female Swiss albino mice were allocated into four distinct groups. Group I served as the control, while Group II received the standard drug diclofenac at a dose of 10 mg/kg. Groups III and IV were administered oral doses of TSE at 50 mg/kg and 100 mg/kg, respectively, for five consecutive days. On the fifth day, chronic inflammation was induced in all animals by a subplantar injection of 0.02 mL of a freshly prepared 2% formalin solution into the right hind paw. Paw thickness was measured with Vernier calipers daily for five days following the formalin injection.

The change in paw thickness was calculated by comparing with the untreated control groups, and % inhibition in inflammation was calculated.

### 4.5. Statistical Analysis

All results are expressed as mean ± SD for each concentration in triplicate. Statistical analysis was performed using analysis of variance (ANOVA), followed by the Tukey–Kramer post-hoc test, with GraphPad Prism software version 7.0 (Boston, MA, USA). Variations with *p* < 0.05 were considered statistically significant.

## 5. Conclusions

In conclusion, TSE demonstrates significant anti-inflammatory activity in an animal model, showing effects that are comparable to those of diclofenac. The dose-dependent response suggests that higher concentrations of TSE may offer enhanced anti-inflammatory benefits. These findings support the potential of TSE as a natural treatment for inflammation. These findings suggest that the studied plant extract can serve as an alternative source of safe anti-inflammatory lead compounds. It is also essential to isolate and characterize the specific phytocompounds responsible for the anti-inflammatory effects of the studied plant extracts. Furthermore, the specific modes of bioactivity on disease conditions such as inflammation and diabetes mellitus, as claimed in traditional medicine, should be established.

## Figures and Tables

**Table 1:** The compounds identified in the *T. siliquosa* extract by HR-LC MS/MS analysis.

Sl. No.	RT	Compound	Molecular Formula	Ionization Mode	*m/z* (Measured)	*m/z* (Calculated)	Diff (ppm)	Fragments
1	1.179	Lotaustralin	C_11_H_19_NO_6_	(M+H)+	264.1333	264.1336	1.01	127.0371, 143.0544, 161.0649, 180.0986, 198.1108, 216.1212, 244.1145
2	1.226	Retronecine	C_8_H_13_NO_2_	(M+H)+	157.1045	157.1051	3.76	156.1013, 130.0855, 138.0545, 152.0691
3	2.097	Gallic acid	C_7_H_6_O_5_	(M−H)−	169.0144	169.0142	−0.83	169.0144
4	4.118	o-Cresol	C_7_H_8_O	(M+HCOO)−	153.0555	153.0557	1.34	153.0555, 107.0494
5	6.473	Quinic acid	C_7_H_12_O_6_	(M−H)−	191.0566	191.0561	−2.58	191.0566
6	6.551	Caffeic acid	C_9_H_8_O_4_	(M−H)−	179.0352	179.0352	2.2	179.0352, 180.0387, 181.0462
7	7.114	Phenethyl salicylate	C_15_H_14_O_3_	(M+Na)+	265.0844	265.0835	−3.15	250.0599, 233.0588, 22.0645, 209.0952, 191.0856, 177.0697, 158.9949, 131.0857
8	7.42	Batatasin II	C_16_H_18_O_4_	(M+Na)+	297.1105	297.1097	−2.71	282.0866, 191.0864
9	7.462	(-)-Epicatechin	C_15_H_14_O_6_	(M+HCOO)−	335.0783	335.0772	−3.04	317.0838, 277.1098, 247.1009, 219.0447, 179.0343, 161.0247, 135.0447, 111.0450
10	8.817	Fabianine	C_14_H_21_NO	(M+H)+	220.1686	220.1679	−17.61	202.1553, 176.1430, 161.1313, 146.0939
11	8.955	Inundatine	C_16_H_23_NO_2_	(M+H)+	262.1784	262.1802	6.67	262.1784, 242.1734, 247.1313
12	9.26	7(14)-Bisabolene−2,3,10,11-tetrol	C_15_H_28_ O_4_	(M+Na)+	272.1993	272.1988	1.01	282.0824, 253.1398, 217.1564, 161.0928, 133.0991
13	9.872	(*E*,*E*,*E*)-Sylvatine	C_24_H_33_NO_3_	(M+Na)+	383.2512	383.246	−1.61	285.1379, 254.1162, 228.1615, 191.1408, 151.1110
14	9.924	Quercetin	C_15_H_10_O_7_	(M+H)+	302.0405	302.0426	5.86	285.0390, 144.1295, 200.1074, 165.0161, 121.0274
15	10.403	Ketosantalic acid	C_15_H_22_O_3_	(M+Na)+	250.1574	250.1569	0.39	233.1060, 213.1253, 195.1126, 165.0674, 153.0161, 143.0851, 132.0519
16	10.518	Gingerol	C_17_H_26_O_4_	(M+H)+	294.1817	294.1831	−2.44	277.1790, 238.0810, 199.1425, 173.1288, 147.1146, 129.0536
17	10.594	Clitorin	C_33_H_40_O_19_	(M−H)−	739.2132	739.2091	−5.54	593.1284, 394.0259, 326.0317, 284.0326, 151.0027
18	10.742	Myricitrin	C_21_H_20_O_12_	(M−H)−	463.0899	463.0882	−3.68	300.0276, 271.0249, 151.0025
19	11.716	Quercitrin	C_21_H_20_O_11_	(M−H)−	447.0949	447.0933	−3.71	284.0327, 227.0352, 151.0048
20	11.795	Luteolin 4′-*O*-glucoside	C_21_H_20_O_11_	(M−H)−	447.0957	447.0933	−5.41	387.0760, 327.0466, 284.0333, 227.0356, 151.0053
21	13.06	1-(2,4,5-Trimethoxyphenyl)-1,2-propanedione	C_12_H_14_O_5_	(M+Na)+	238.0847	238.0841	−1.3	246.0507, 231.0271, 217.0793, 190.9943, 163.0033, 131.0812
22	13.972	Guaiazulene	C_15_H_18_	(M+H)+	198.1397	198.1409	6.33	184.1249, 173.1335, 157.0994, 143.0857, 129.0689
23	17.176	Coumaric acid	C_17_H_14_N_2_O_7_	(M+H)+	361.092	361.0928	2.01	324.0481, 296.0649, 280.0580, 265.0385, 252.0707, 221.0573, 207.0398, 193.0619, 164.0481
24	17.426	Panaxytriol	C_17_H_26_O_3_	(M+Na)+	301.1756	301.1774	6.04	252.0552, 223.1464, 185.1351, 159.1143, 133.0997
25	19.164	Colnelenic acid	C_18_H_28_O_3_	(M−H)−	291.1963	291.1966	0.77	291.1965, 205.8387, 165.1275
26	19.736	α-Corocalene	C_15_H_20_	(M+H)+	202.1663	202.1672	4.15	186.1214, 173.1324, 159.1141, 145.0992, 131.0829, 121.0992
27	20.161	Citronellyl hexanoate	C_16_H_30_O_2_	(M+Na)+	278.2178	278.2172	−2.22	277.2155, 209.1525
28	24.474	Euphornin	C_33_H_44_O_9_	(M+Na)+	584.2983	584.2985	0.5	547.2653, 505.2219, 460.2219, 433.2323, 372.1422, 262.1336, 146.1020

**Table 2:** In silico docking analysis of major compounds against selected targets lipoxygenase and cyclooxygenase 2 (kJ/mol).

Compounds	LOX	COX-2
Quercetin	−6.625 ± 0.31	−7.875 ± 0.56
Ketosantalic acid	−4.975 ± 0.21	−6.725 ± 0.50
Coumaric acid	−5.875 ± 0.48	−6.425 ± 0.46
Quinic acid	−6.375 ± 0.43	−6.450 ± 0.62
Gallic acid	−5.825 ± 0.17	−7.400 ± 0.29
Myricitrin	−7.075 ± 0.22	−7.475 ± 0.20
Quercitrin	−5.150 ± 0.07	−6.250 ± 0.21
Luteolin 4′-*O*-glucoside	−6.075 ± 0.68	−6.625 ± 0.59
(-)-Epicatechin	−6.475 ± 0.30	−6.625 ± 0.31
Diclofenac	−7.200 ± 0.61	−8.125 ± 0.27

**Table 3:** Changes in the paw thickness of mice induced inflammation with carrageenan and protective efficacy by *T. siliquosa* leaf methanolic extract.

Treatment Group	Paw Thickness		Increase in Paw Thickness	Percentage Inhibition with Respect to Control (%)
	Initial	Final		
Control	1.78 ± 0.18	2.33 ± 0.29	0.55 ± 0.07	0.0
Diclofenac	2.00 ± 0.20	2.18 ± 0.19	0.18 ± 0.04 **	67.3 ± 2.3
Drug (50 mg)	1.88 ± 0.10	2.13 ± 0.17	0.26 ± 0.11 *	53.0 ± 2.5
Drug (100 mg)	1.90 ± 0.08	2.12 ± 0.26	0.22 ± 0.14 **	60.0 ± 1.8

* indicates significant variation with respect to the control (*p* < 0.05); ** indicates higher significant variation with respect to the control (*p* < 0.01).

**Table 4:** Changes in the paw thickness of mice induced inflammation with formalin and protective efficacy by *T. siliquosa* leaf methanolic extract.

Treatment Group	Paw Thickness			% Inhibition with Respect to Control	
	Initial	Day 5	Day 10	Day 5	Day 10
Control	2.05 ± 0.12	3.78 ± 0.17	2.86 ± 0.08	0.00	0.00
Diclofenac	2.25 ± 0.18	3.19 ± 0.13 *	2.44 ± 0.15 **	46.17 ± 2.1	76.89 ± 2.8
Drug (50 mg)	2.19 ± 0.11	3.49 ± 0.10 *	2.59 ± 0.11 *	24.74 ± 3.2	50.48 ± 2.3
Drug (100 mg)	2.15 ± 0.09	3.07 ± 0.14 *	2.44 ± 0.12 **	47.04 ± 1.9	64.72 ± 2.2

* indicates significant variation with respect to the control (*p* < 0.05); ** indicates higher significant variation with respect to the control (*p* < 0.01).

## Data Availability

Data may be made available upon valid request.

## Supplementary Materials

The following supporting information can be downloaded at: https://www.mdpi.com/article/10.3390/molecules29204800/s1, Supplementary Figure S1. MS Spectrum (a) and MS/MS spectrum (b) of the following compounds. Supplementary Figure S2. Chemical structures of major bioactive compounds in *T. siliquosa* extract.
