# Supplementary material for: Methanol Extract of Thottea siliquosa (Lam.) Ding Hou Leaves Inhibits Carrageenan- and Formalin-Induced Paw Edema in Mice
Source: Molecules. 2024 Oct 11;29(20):4800. doi: 10.3390/molecules29204800 (PMC11510445; doi:10.3390/molecules29204800)

Supplementary Figure S1. MS Spectrum (a) and MS/MS spectrum (b) of the following compounds;

(1) Lotaustralin

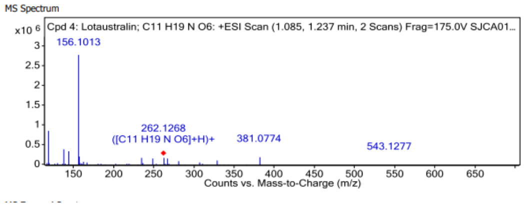

(a)

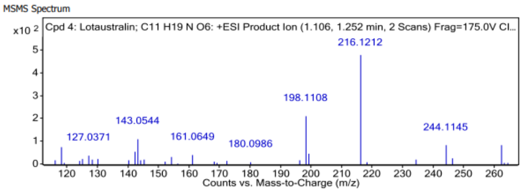

(b)

(2) Retronecine

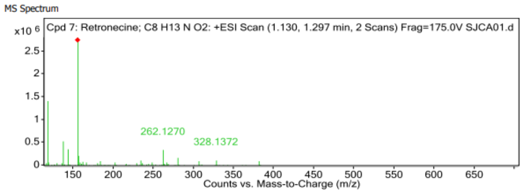

(a)

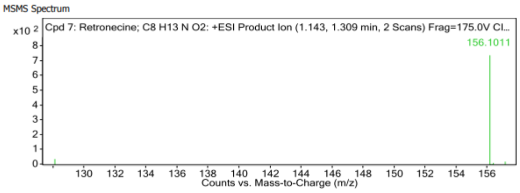

(b)

(3) Phenethyl salicylate

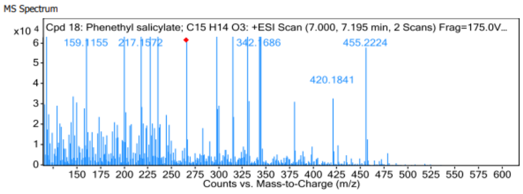

(a)

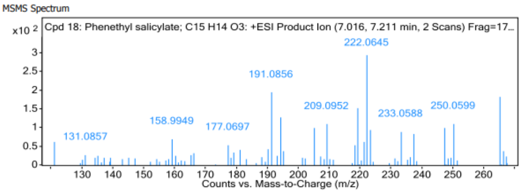

(b)

(4) Fabianine

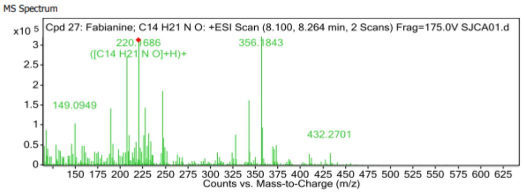

(a)

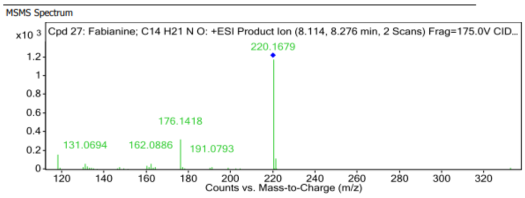

(b)

(5) Inundatine

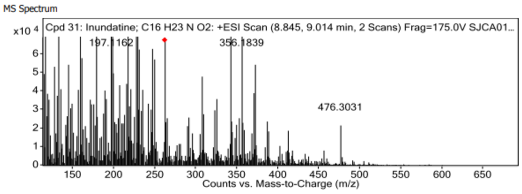

(a)

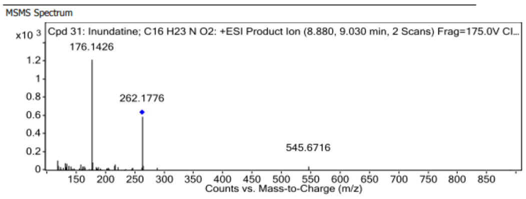

(b)

(6) 7(14)-Bisabolene-2,3,10,11-tetrol

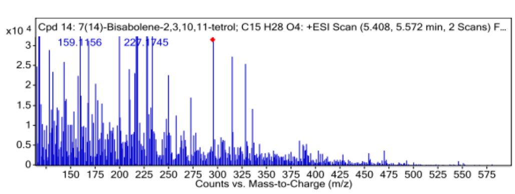

(a)

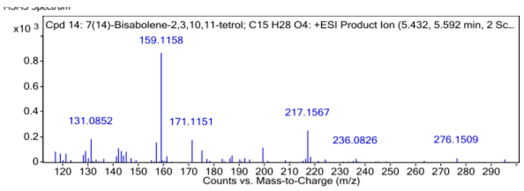

(b)

(7) (E,E,E)-Sylvatine

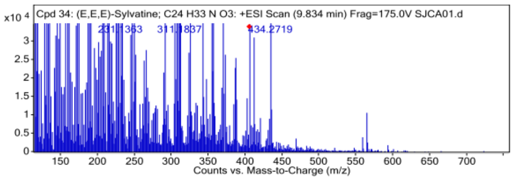

(a)

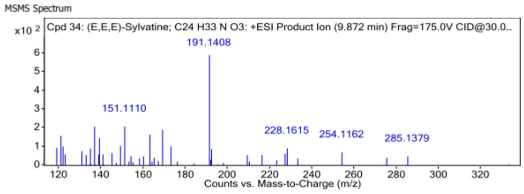

(b)

(8) Quercetin

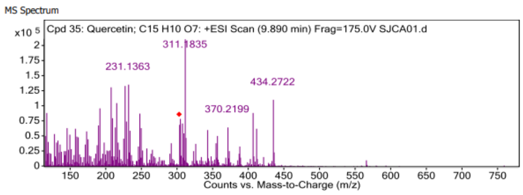

(a)

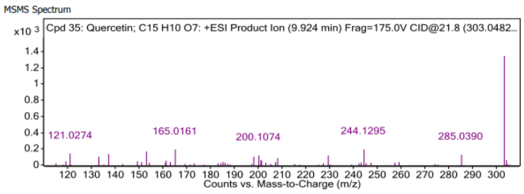

(b)

(9) Ketosantalic acid

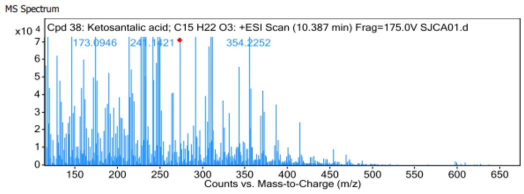

(a)

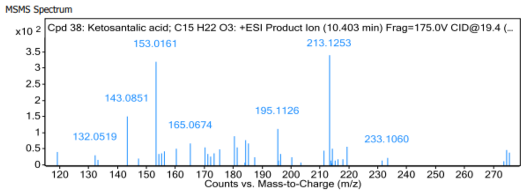

(b)

(10) Gingerol

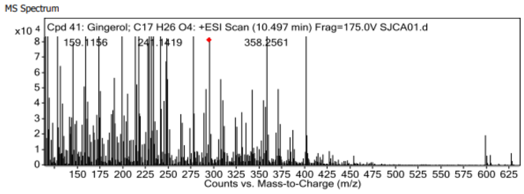

(a)

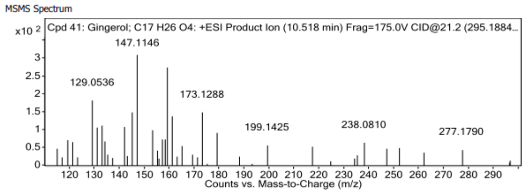

(b)

(11) 1-(2,4,5-Trimethoxyphenyl)-1,2-propanedione

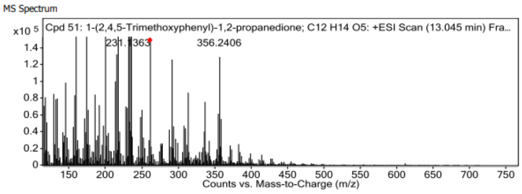

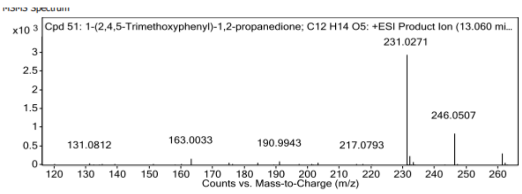

(a) (b)

(12) Guaiazulene

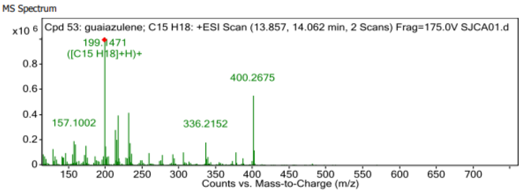

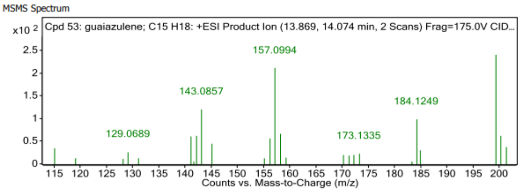

(a) (b)

(13) Coumeroic acid

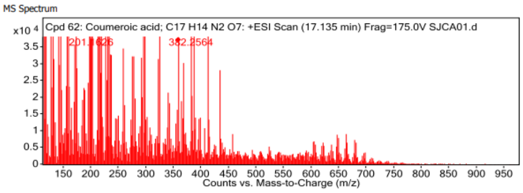

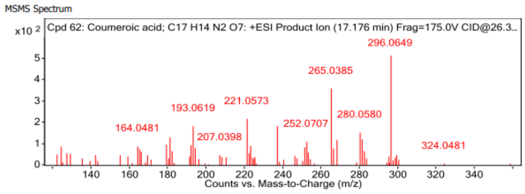

(a) (b)

(14) Panaxytriol

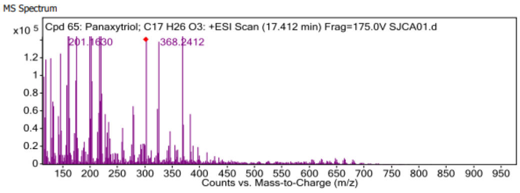

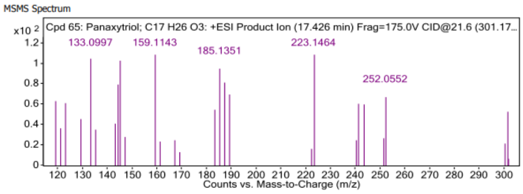

(a) (b)

(15) alpha-Corocalene

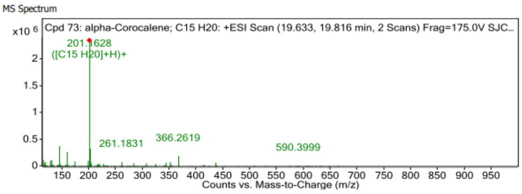

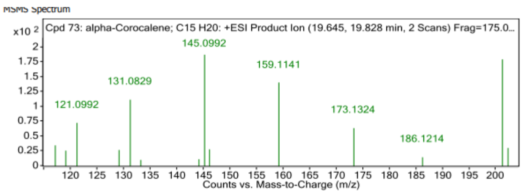

(a) (b)

(16) Citronellyl hexanoate

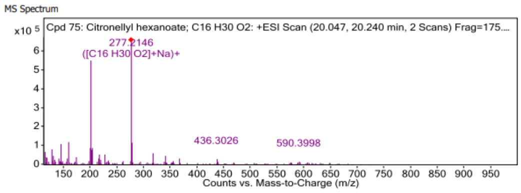

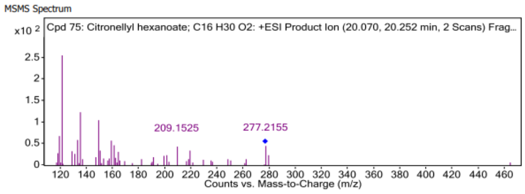

(a) (b)

(17) Euphornin

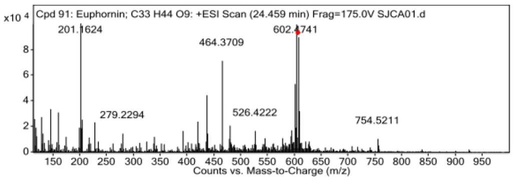

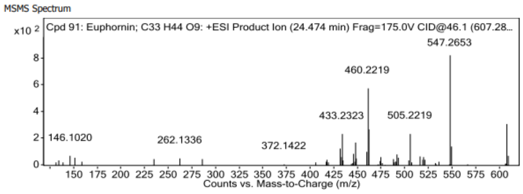

(a) (b)

(18) Quinic acid

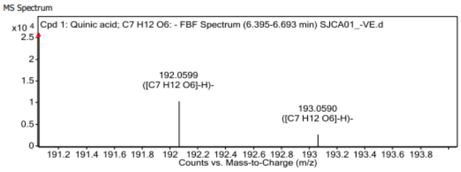

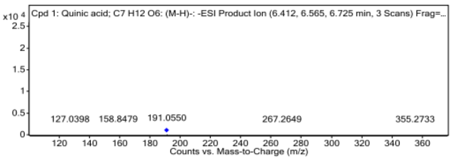

(a) (b)

(19) Quercitrin

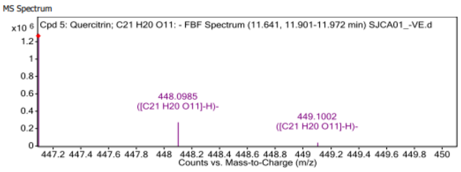

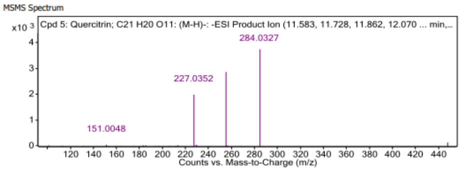

(a) (b)

(20) Luteolin 4'-O-glucoside

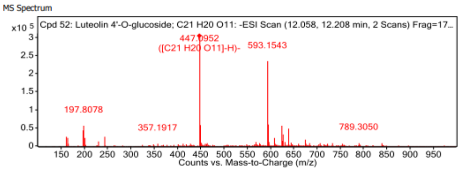

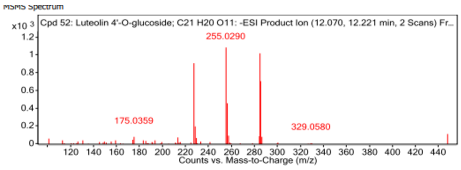

(a) (b)

(21) Colnelenic acid

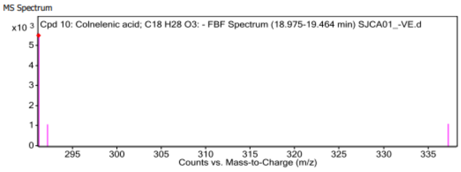

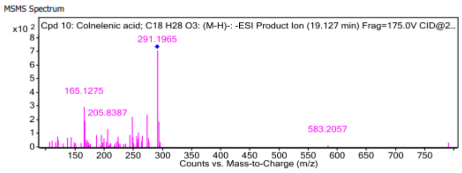

(a) (b)

(22) Gallic acid

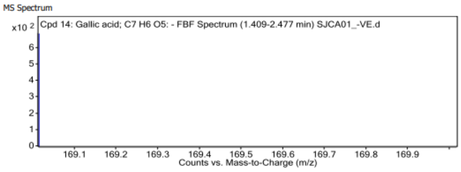

(a)

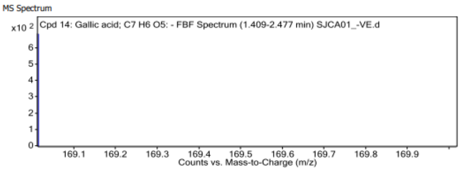

(b)

(23) o-Cresol

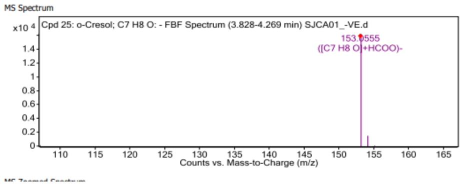

(a)

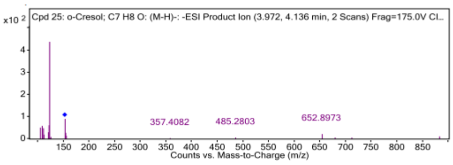

(b)

(24) Caffeic acid

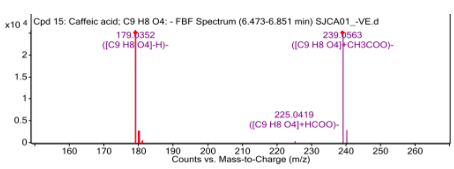

(a)

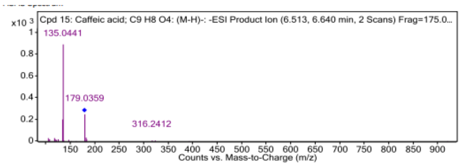

(b)

(25) Clitorin

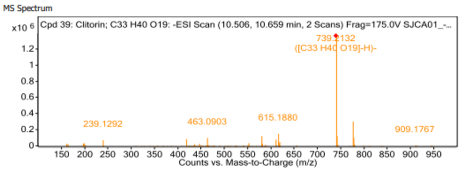

(a)

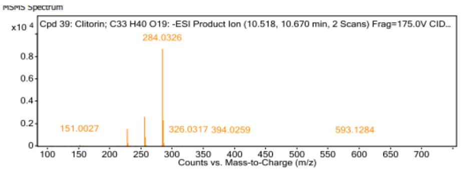

(b)

(26) Myricitrin

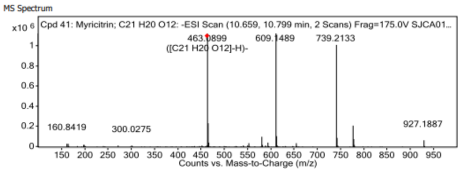

(a)

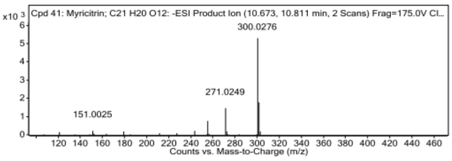

(b)

(27) (-)-Epicatechin

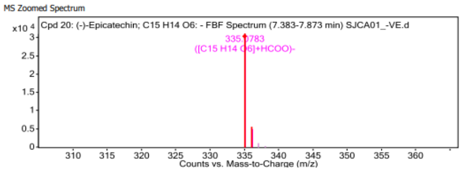

(a)

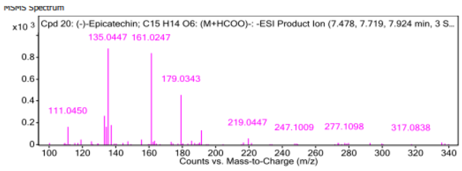

(b)

(28) Batatasin II

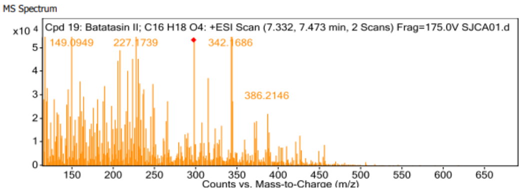

(a)

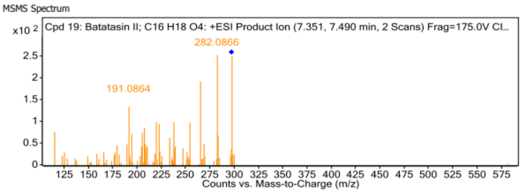

(b)

Supplementary Figure S2. Major bioactive compounds in *T. siliquosa* extract

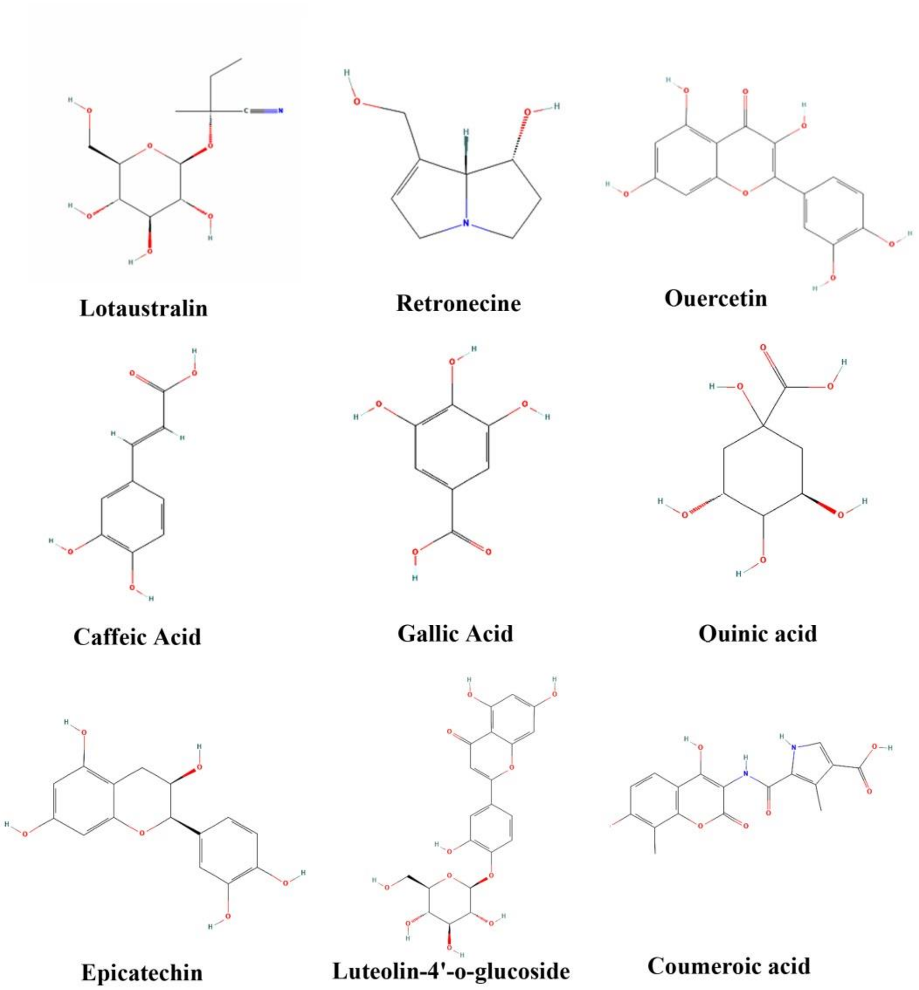

Supplement: Supplementary file 1 [file molecules-29-04800-s001.zip › molecules-3162860-supplementary.pdf]
